# Self-Assessment of INTERHEART Risk Stratification among the Middle-Aged Community in Malaysia

**DOI:** 10.3390/nu15102382

**Published:** 2023-05-19

**Authors:** Siew-Keah Lee, Ang-Lim Chua, Clement Heng Yew Fong, Brian Cong Hao Ban, Wen Ling Ng, Jing Feng Kong, Yik-Ling Chew, Kai Bin Liew

**Affiliations:** 1 M. Kandiah Faculty of Medicine and Health Sciences, Universiti Tunku Abdul Rahman, Kajang 43000, Malaysia; 2 Faculty of Medicine, Universiti Teknologi MARA, Sungai Buloh 47000, Malaysia; 3 Faculty of Pharmaceutical Sciences, UCSI University, Kuala Lumpur 56000, Malaysia; 4 Faculty of Pharmacy, University of Cyberjaya, Cyberjaya 63000, Malaysia

**Keywords:** non-laboratory-based cardiovascular disease risk score, INTERHEART, middle-aged, CVD risk, lifestyle, dietary pattern, physical activity, psychosocial status

## Abstract

**Research background and Objectives:** Age is an independent risk factor for cardiovascular disease (CVD), but CVD risk factors are preventable, and lack of awareness of its risk factors is a contributing factor to CVDs. Middle-aged people may be more likely to engage in unhealthy lifestyle behaviours which can increase the risk of CVD. Health self-assessment is crucial for early detection and management of health issues and early lifestyle intervention for better personalised health management. This study aims to determine the self-assessment of INTERHEART risk classification among the middle-aged community in Malaysia. **Method:** Local community members aged 40–60 years and who are currently residing in Malaysia were recruited via non-randomised sampling. Sociodemographic characteristics and dietary pattern related to salt, fibre, fat (deep fried/snacks), poultry/meat intakes, and other cardiovascular risk factors (waist-hip ratio, medical history related to diabetes/hypertension, history/exposure of tobacco use, psychosocial status, and level of physical activity) were assessed; INTERHEART risk scores were then computed and stratified into low, medium and high risks. **Results:** Approximately 45% (*n* = 273/602) of middle-aged respondents in Malaysia are at moderate-to-high risk of cardiovascular events, with men being more likely to develop CVD compared to women. The results of the survey indicated that poultry/meat intake (61%), physical inactivity (59%), and second-hand smoke (SHS) exposure (54%) are the most prevalent risk factors among the respondents. One-third of the respondents consumed excessive salty food and deep fried foods/snacks/fast food, and only one-third of them consumed vegetables/fruits at a recommended level. It is worrying that about a quarter of the respondents felt several periodical/permanent stresses and even felt sad/blue/depressed for two weeks or more in a row. Males, labour workers, and those with lower educational levels are more likely to develop CVD events. **Conclusions:** This study found that 45% of the middle-aged respondents were having moderate-to-high risk for cardiovascular events with multiple risk factors related to unhealthy lifestyle habits and environmental factors. In addition to non-modifiable factors such as gender and age, sociodemographic factors, i.e., educational level and occupation, are equally important factors to determine CVD risk. Overall, the findings of this study emphasize the clinical relevance of assessing multiple factors in the determination of CVD risks for early prevention and management of cardiovascular diseases.

## 1. Introduction

Cardiovascular disease (CVD) is a major global health issue, causing significant morbidity and mortality worldwide, including in Malaysia [1]. Unfortunately, it is often asymptomatic and can go undetected until it has progressed to an advanced stage, resulting in higher rates of morbidity and mortality. While it is widely recognized that the elderly (>65 years old) are at greater risk of developing CVD, it is crucial to take preventive measures at an early age. Early prevention is the key in combating CVD. Lifestyle modifications such as having a healthy diet, regular exercise, and avoiding tobacco use can reduce the risk of developing CVD. Furthermore, regular medical check-ups with a healthcare provider can identify risk factors and enable early intervention. Identifying and managing risk factors such as high blood pressure, diabetes, and high cholesterol levels can also reduce the risk of developing CVD [1,2].

Health self-assessment plays crucial roles in the early detection of diseases with no noticeable symptoms. The process of health self-assessment involves evaluating an individual’s health/medical condition, dietary habits, stress level, physical level, etc. Common point-of-care tools are typically used for assessing body temperature, blood pressure, cholesterol, glucose and oxygen saturation levels, etc. It is worth mentioning that these point-of-care devices, especially the portable pulse oximeter and thermometer, served as important health self-assessment tools over the COVID-19 pandemic when the healthcare systems were overwhelmed [3]. Other important indices such as lifestyle habits which include smoking, physical inactivity, stress levels, and poor diet are equally as important for a more complete health assessment of CVD risks as well as general health [1,4].

CVD risk stratification is the process of assessing an individual’s risk of developing cardiovascular diseases based on their health status and medical history. This assessment helps to identify people who are at a high risk of developing CVD and to take preventative measures to reduce their risk. CVD stratification involves the evaluation of various risk factors such as age, gender, family history of CVD, blood pressure, cholesterol levels, smoking status, diabetes, and obesity. Based on the results of this assessment, individuals can be categorized into different risk categories, including low, intermediate, or high risk. For individuals at high risk, interventions such as lifestyle modifications (e.g., regular exercise, a healthy diet, and weight management), medication, and medical procedures may be recommended to reduce their risk of developing CVD [4].

There are various CVD risk stratification tools available, and the more commonly used tools include Framingham Risk Score (FRS), Reynolds Risk Score (RRS), risk score using QRESERCH database (QRISKS), ACC/AHA Pooled Cohort Equations, and the Systematic Coronary Risk Evaluation (SCORE) [4]. The FRS considers factors such as age, gender, total cholesterol, HDL cholesterol, blood pressure, smoking, and diabetes status to estimate the 10-year risk of developing CVDs. PRS is similar to the FRS but with the addition of measuring plasma C-reactive protein. QRISKS uses information such age, ethnicity, smoking status, blood pressure, cholesterol levels, body mass index (BMI), and family history of CVD to estimate the 10-year risk of developing CVD. The ACC/AHA Pooled Cohort Equations take into account factors such as age, gender, total cholesterol, HDL cholesterol, blood pressure, smoking, and diabetes status. SCORE estimates the 10-year risk of fatal CVD based on factors such as age, gender, smoking status, total cholesterol, and systolic blood pressure. In Malaysia, the FRS is adopted in the Malaysian Clinical Practice Guidelines on the Primary and Secondary Prevention of Cardiovascular Disease, 2017. These tools all require measuring blood markers and complex algorithms for interpretation and, thus, are not suitable for patient self-assessment of CVDs risks. In addition, psychosocial factors and physical activity are not taken into account for the risk prediction.

The non-laboratory INTERHEART risk score was shown to accurately predict an individual’s risk of CVD based solely on their clinical history and simple physical measurements [5,6]. Various studies have concluded that the INTERHEART tool provided similar accuracy to other assessment tools that involved laboratory measurement of blood markers for predicting CVD, e.g., FRS, SCORE, WHO/ISH [7,8], and it has been showed that the INTERHEART risk score is a reliable predictor of CVD risk among Malaysians and other populations [6,9]. This makes it an ideal choice for healthcare providers working in low-resource settings and even as a patient self-assessment tool for CVD risk stratification.

This present study examined the use of INTERHEART risk stratification for CVD risk self-assessment among the middle-aged population in Malaysia. The data collected are all self-reported and include basic medical history, present waist-to-hip ratio (with guidelines provided within the survey), smoking habits, psychosocial status, and dietary habits (including intake of salt, fruits, vegetables, fried food, fast food, snacks, and poultry/meat), as well as their level of physical activity. The study aimed to determine demographic groups that have a higher probability of developing cardiovascular disease (CVD) and to educate participants on the significance of early detection of CVD risks in order to implement tailored lifestyle changes that can lead to improved personal health management.

## 2. Subjects and Methods

### 2.1. Study Design and Setting

This cross-sectional survey was conducted during November 2022–January 2023 via convenience and snowball sampling methods. This study was approved by the Institutional Scientific and Ethical Review Committee (U/SERC/236/2022) and conducted in accordance with the code of ethics.

### 2.2. Survey Instrument

The questionnaire was prepared in English, and the forward and backward translation technique was used for the questionnaire preparation in Bahasa Malaysia and Chinese (Simplified).

A preliminary study was carried out involving 30 participants who completed the questionnaire. The findings showed high levels of internal consistency, with alpha Cronbach values exceeding 0.8. All data obtained from the preliminary study were excluded from the actual data analysis.

The first section of the questionnaire was related to sociodemographic profile. The participants’ self-report on their occupation type is based on the level of physical activity involved during working hours. Occupations that typically require high levels of physical activity, such as labour workers, farmers, and those working in construction sites or factories, are classified as having high physical activity. On the other hand, those who work in office settings where sedentary activities are common, such as desk jobs, are classified as having low physical activity. The respondents were required to self-assess their health status according to a series of questions related to INTERHEART score evaluation in the second section of the questionnaire. This scoring was based on risk factors including age and gender, family history of heart attack, smoking status, second-hand smoke (SHS) exposure, diabetes mellitus (DM) status, hypertension status, waist-to-hip ratio (WHR), stress level, depression, dietary habits, and physical activity status. Each positive risk factor was assigned a specific score, resulting in a total score ranging from 0 to 48. Higher scores indicated greater risk. Previous studies established the score categories as low risk (scores between 0 and 9), moderate risk (scores between 10 and 15), and high risk (scores between 16 and 48) [5,6].

### 2.3. Participation Eligibility

Eligible participants with ages between 40 and 60 years old who reside in Malaysia and understand English, Bahasa Malaysia, or Chinese (Simplified) were invited for survey participation.

### 2.4. Survey Invitation and Informed Consent

Hard copies of the questionnaire were disseminated among the public through convenience sampling. The completed questionnaires were collected on the same day of distribution. The online questionnaire was sent to personal contacts via emails, short message service (SMS), and mobile messenger apps. The same invitation was also posted to various online social media platforms to call for public participation. The potential respondents were encouraged to disseminate the same invitation to their contacts.

All the participants were asked to provide informed consent before proceeding to the questionnaire. The participants can withdraw from the study by stopping answering the questions.

### 2.5. Data Analysis

The statistical analysis was performed using Statistical Package for Social Science (SPSS) version 22.0. Categorical data were expressed as frequency and percentage, while continuous data were expressed as mean and standard deviation (mean ± SD). One-way ANOVA followed by Tukey post hoc or independent *t*-test was used to test the difference of the means of independent groups. Linear regression was used to determine the factors associated with INTERHEART mean scores. Chi-square test was used to test the association among categorical variables. A *p*-value of less than 0.05 was considered statistically significant.

## 3. Results

**Figure 1:**
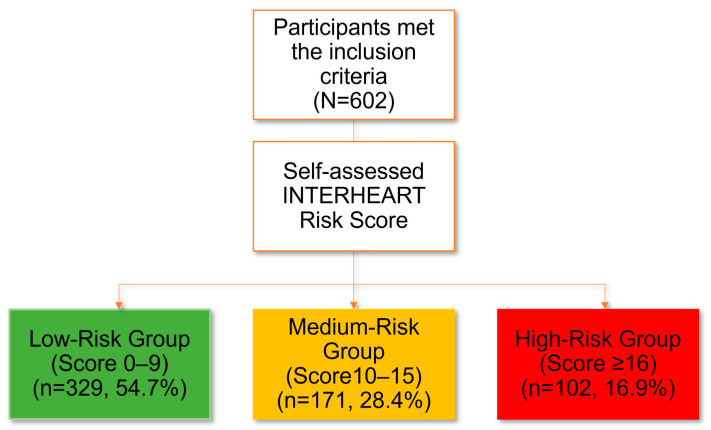
CVD risk stratification among the respondents (N = 602).

**Figure 2:**
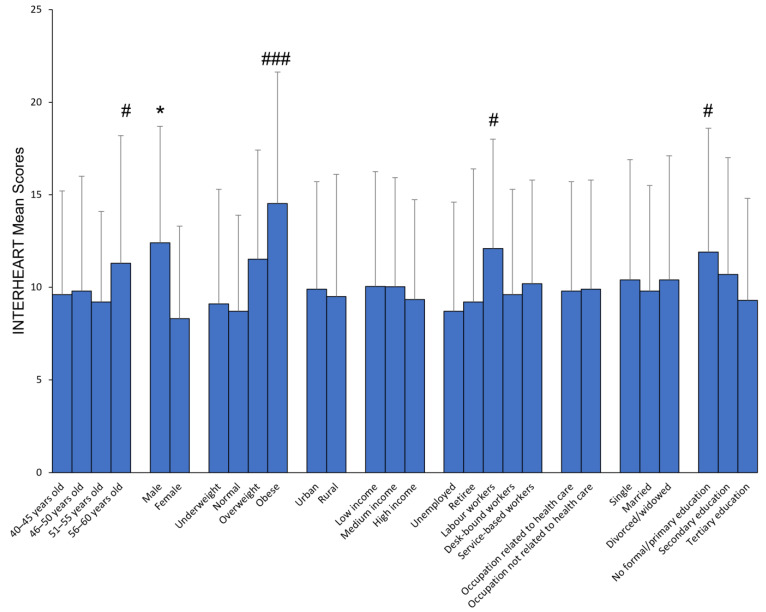
Sociodemographic and INTERHEART mean scores. * *p* < 0.05 compared to counterpart, independent *t*-test; # *p* < 0.05, ### *p* < 0.001 compared to counterparts, ANOVA.

Figure 1 shows the CVD risk stratification among the respondents (N = 602). Figure 2 and Table 1 display how sociodemographic factors are distributed, along with the corresponding mean score of the individuals’ INTERHEART score for each factor. It was found that individuals who are older, male, have a high BMI, engage in labour-based occupation, have a low educational level, and are not aware of self-assessment tools are more likely to have a higher risk of CVD compared to those who do not have these factors. It is noteworthy that exposure to information in posters or brochures about CVD did not seem to have a significant impact on the INTERHEART mean score. Table 2 shows the risk factors associated with INTERHEART mean scores. Linear regression test shows that gender, BMI, and educational level are associated with INTERHEART mean scores.

Table 3 displays the characteristics of the participants according to INTERHEART risk factors, stratified by gender. The results of the survey indicate that meat/poultry intake (61%), physical inactivity (59%), and second-hand smoke (SHS) exposure (54%) are the most prevalent risk factors among the respondents. While one-third of the respondents consumed excessively salty food and deep fried foods/snacks/fast food, only one-third of them consumed vegetables/fruits at a recommended level. It is worrying that about a quarter of the respondents felt several periodical/permanent stresses and even felt sad/blue/depressed for two weeks or more in a row.

It was discovered that males were more prone to being active smokers, having prolonged exposure to SHS, having a medical history of diabetes and hypertension, possessing a high waist-to-hip ratio, and consuming more deep-fried foods, snacks/fast foods, and meat/poultry compared to their counterparts. On the other hand, females tend to be more sedentary. Overall, male respondents are at higher risk of developing CVD, with 28.6% being classified under high risk compared to 9.1% among the female respondents.

Table 4 shows the characteristics of the participants according to INTERHEART risk factors, stratified by occupation. It was revealed that labour workers were likely to be active/former smokers, less likely to have sufficient amount of fruits/vegetables, and have longer exposure duration of SHS compared to the desk-bound workers. Further Chi-square analysis showed that the educational level of those labour workers is lower than of the desk-bound workers (*p* < 0.001, Table 5). 

## 4. Discussion

The key findings of this study show that a significant proportion of middle-aged individuals in Malaysia, namely 28% and 17%, are at moderate-to-high risk for CVD events. Men were found to have a higher likelihood of developing cardiovascular disease (CVD) compared to women. The study’s results showed strong agreement with the risk stratification conducted in a previous study involving 14,863 Malaysian participants aged 40–65 years old, in which 29% and 23% of the participants were at moderate-to-high risk of CVD events [8], indicating the reliability and validity of the current findings. The findings of this study have identified that excessive meat/poultry intake, physical inactivity, and second-hand smoke exposure are the most prevalent behavioural risk factors in addition to significant sociodemographic risk factors including being male, having lower educational levels, and having a higher BMI. Labour workers were also identified as a group at increased risk for CVD events. Those not aware of self-assessment tools that help to predict CVD risk and those who did not undergo annual medical check-ups are prone to have higher INTERHEART mean scores compared to their counterparts. These results emphasize the importance of addressing modifiable behavioural risk factors such as diet, physical activity, and smoking, as well as targeting high-risk sociodemographic groups with tailored interventions.

The data showed that gender is a crucial determinant of CVD risk, and this highlights the importance of considering gender-specific factors in CVD prevention, diagnosis, and treatment. While genetic predisposition contributes to CVD risk in males, lifestyle factors also play a significant role. Specifically, men are more likely to engage in high-risk behaviours such as smoking and consuming alcohol frequently [2]. This study reinforces these gender-based disparities in Malaysia, demonstrating that middle-aged men are at greater risk for CVD due to their behaviours, including frequent consumption of deep-fried foods, snacks, and fast food, as well as meat and poultry; and these may contribute to the higher prevalence of central abdominal obesity (reflected via waist-to-hip ratio) among men, compared to women. The findings of this study also indicate that the active and former smoking prevalence among men are approximately 7-fold higher, and their SHS exposure duration is 2.4-fold higher than women. Additionally, the prevalence of diabetes and hypertension is significantly higher in men than in women, all these factors leading to 28.6% of men being classified as high risk (score ≥ 16) of CVD. Overall, the findings of this study emphasize the importance of addressing gender-specific factors in CVD risk-reduction interventions, particularly for middle-aged men in Malaysia who appear to be at greater risk than their female counterparts.

The findings of this study indicate that a significant majority of the respondents reported consuming meat or poultry at least twice daily. While previous studies have often focused on monitoring the intake of salt, fat, and fibre in the participants’ diets, meat and poultry consumption has been a largely neglected aspect of dietary assessment. Therefore, this study brings attention to the importance of including meat and poultry intake as a key factor in evaluating dietary habits and highlights the need for further research to investigate its potential impact on health outcomes, especially risk of developing CVD.

Numerous studies have indicated a link between omitting meat from one’s regular dietary habits with a decreased likelihood of developing cardiovascular disorders. Populations that follow a predominantly plant-based diet have been observed to have lower rates of heart disease and other chronic illnesses compared to those who consume meat regularly [10,11,12]. Meat and poultry, with their high content of saturated and trans fats, can raise LDL cholesterol levels of consumers and increase the risk of CVD. Furthermore, meat and poultry are rich in heme iron, which can increase oxidative stress and inflammation in the body, leading to an increased risk of CVD [13]. Conversely, plant-based diets are typically higher in fibre, vitamins, and minerals, while being lower in saturated and trans fats. These dietary factors can help to reduce inflammation, improve blood pressure, and lower cholesterol levels, all of which are important factors in preventing CVD.

The survey results revealed that almost 60% of middle-aged participants reported engaging in primarily sedentary activities or mild exercise that demands minimal exertion. Middle-aged adults often have demanding work and family responsibilities which leave them with limited time and energy for physical exercise. A previous survey attributed physical inactivity among Malaysian young adults to a lack of individual motivation, rather than environmental barriers or inadequate knowledge of the benefits of physical activity [14].

The Malaysian government has made significant strides in promoting an active lifestyle by increasing public recreational and sports facilities, awareness programs, and tax incentives to individuals who sign up for gym memberships and purchase sports equipment [1]. Despite the numerous national intervention programs, their efforts often fall short of achieving sustained behaviour change. Governmental programs often fail to sustain long-term interest and engagement, as they fail to address the underlying factors that influence people’s behaviour over the long term, such as social norms, cultural beliefs, and individual motivations. Sustaining a behaviour change requires ongoing support, encouragement, and reinforcement, which may not always be available through these programs. Thus, it is essential to employ techniques that foster sustained motivation and promote health-promoting behaviour, such as self-regulatory skills in setting realistic goals and utilizing appropriate reward systems. This present study proposes to integrate physical activity into daily routines based on personalized interests and goals. This approach acknowledges that individuals have unique preferences and motivations for physical activity and tailors interventions accordingly. By incorporating activities that align with an individual’s interests and values, the likelihood of adherence and sustained engagement is increased. Secondly, incorporating the use of fitness trackers/wearable devices has been shown to encourage physical activity engagement. These devices provide individuals with real-time feedback on their progress, which can serve as a source of motivation and reinforce their behaviour change. Additionally, the social connectivity and accountability features of these devices can foster a sense of community and support, which is crucial for sustaining behaviour change over time.

It is typical to link the amount of physical activity someone engages in with their job, and often we see that manual laborers tend to have higher levels of physical activity compared to those who work at a desk all day. Surprisingly, the findings of this present study showed that the degree of physical activity during leisure time is comparable between labour and desk-bound workers. This study also found that those labour workers had a significantly higher CVD risk, as indicated by their INTERHEART score, than desk-bound workers. This increased risk may be associated with other factors such as diet and lifestyle habits, educational level, and perhaps the degree of awareness/knowledge related to CVD risk. Research has consistently demonstrated that individuals who have higher levels of education and possess greater knowledge related to CVD risk factors are less likely to be at risk of developing CVD [15]. This relationship between education, knowledge, and CVD risk is thought to be due to a number of factors, including a greater understanding of healthy lifestyle behaviours, increased access to healthcare resources, and a greater ability to make informed decisions regarding one’s health. Furthermore, education and knowledge related to CVD risk can empower individuals to take an active role in managing their health, making positive lifestyle changes, and seeking appropriate medical care when needed. Therefore, promoting education and knowledge related to CVD risk is an important public health strategy for reducing the burden of CVD.

Out of the total respondents, only 8.8% (*n* = 53) smoked regularly in the past 12 months. However, the results indicate that up to 54% of the participants were exposed to SHS for one or more hours per week, which is a matter of concern. On average, the respondents were exposed to SHS for 2.77 h per week. Interestingly, the study found that men were exposed to SHS significantly more than women. These findings highlight the need for immediate action to reduce SHS exposure among the general population, especially among men. According to the Global Adult Tobacco Survey-Malaysia (GATS-M), which assessed the prevalence of tobacco use and exposure to SHS smoke among the non-smoking Malaysians aged 15 years and above, a staggering 61.8% (approximately 5.58 million non-smokers) were found to be exposed to SHS at least once a month either in their homes or workplaces [16]. This present study showed that more than half of the 40–60-year-old respondents (*n* = 325, 54%) were exposed to SHS at least one or more hours per week. It is important to emphasize that any level of exposure to SHS is not safe, and even a short exposure to it can lead to damage to the cardiovascular system [17,18].

Second-hand smoke (SHS), also known as passive smoke or environmental tobacco smoke, refers to the smoke that is exhaled by a smoker or that comes from the burning end of a cigarette, cigar, or pipe. SHS smoke contains many of the same harmful chemicals that are found in the smoke that the smoker inhales, including carbon monoxide, nicotine, and tar [19]. Exposure to SHS has been linked to an increased risk of CVD because it can damage the lining of blood vessels and lead to the development of atherosclerosis. Atherosclerosis, in turn, can restrict blood flow to the heart, brain, and other organs, which can increase the risk of heart attack, stroke, and other cardiovascular problems. It has been reported that non-smokers who breathe SHS increase their risk of developing heart disease by 25–30% and increase the risk of stroke by 20–30% [18,19,20]. This finding highlights the urgent need for more comprehensive tobacco control policies in Malaysia to protect non-smokers from the harmful effects of SHS. In addition, it is crucial to raise public awareness about the dangers of SHS and promote smoke-free environments to reduce the burden of tobacco-related diseases and deaths in Malaysia.

Health self-assessment tools may be useful for individuals to evaluate their health status and identify potential risk factors or health concerns. However, it is undeniable that the reliability of health self-assessment depends on several factors, such as the accuracy of the information provided by the individual, the validity of the questions and tools used for self-assessment, and the individual’s level of health literacy and understanding of their own health status. Therefore, it is important to remember that health self-assessment should not replace regular check-ups and evaluations by a healthcare professional, and individuals should seek medical advice and guidance from a healthcare professional for any health concerns.

## 5. Conclusions and Perspective

This study found that 45% of middle-aged respondents in Malaysia are at moderate-to-high risk for cardiovascular events, with unhealthy lifestyle habits and environmental factors being the contributing factors to CVD. Poultry/meat intake is the most prevalent risk factor, followed by physical inactivity and SHS exposure. In addition to non-modifiable factors such as gender and age, sociodemographic factors, i.e., educational level and occupation, are equally important factors to determine CVD risk. Overall, these findings of this study emphasize the clinical relevance of assessing multiple factors in the determination of CVD risks for early prevention and management of cardiovascular diseases.

## Figures and Tables

**Table 1:** Sociodemographic and INTERHEART mean scores, N = 602.

	*n* (%)	INTERHEART Risk Mean Score (SD)
**Age Group**		
40–45	213 (35.4)	9.6 (5.6)
46–50	143 (23.8)	9.8 (6.2)
51–55	127 (21.1)	9.2 (4.9)
56–60	119 (19.8)	11.3 (6.9) ^#^
**Gender**		
Male	241 (40.0)	12.4 (6.3) *
Female	361 (60.0)	8.3 (5.0)
**BMI**		
Underweight	32 (5.3)	9.1 (6.2)
Normal	372 (61.8)	8.7 (5.2)
Overweight	147 (24.4)	11.52 (5.9)
Obese	51 (8.5)	14.53 (7.1) ^###^
**Area of residence**		
Urban	552 (91.7)	9.9 (5.8)
Rural	50 (8.3)	9.5 (6.6)
**Monthly household income**		
Low (RM < 4850)	214 (35.5)	10.05 (6.2)
Medium (RM 4850–10,970)	261 (43.4)	10.03 (5.9)
High (RM > 10,970)	127 (21.1)	9.34 (5.4)
**Nature of occupation based on level of physical activity**		
Unemployed	45 (7.5)	8.7 (5.9)
Retiree	58 (9.6)	9.2 (7.2)
Manual/labour workers/requires high physical activity during working hours	55 (9.1)	12.1 (5.9) ^#^
Office and desk-bound workers/requires less physical activity during working hours	288 (47.8)	9.6 (5.7)
Service-based/requires moderate physical activity during working hours	156 (25.9)	10.2 (5.6)
**Occupation related to health care**		
Yes	52 (8.6)	9.8 (5.9)
No	550 (91.4)	9.9 (5.9)
**Marital status**		
Single	108 (17.9)	10.4 (6.5)
Married	462 (76.7)	9.8 (5.7)
Divorced/widowed	32 (5.3)	10.4 (6.7)
**Educational level**		
No formal/primary education	36 (6.0)	11.9 (6.7) ^#^
Secondary education	196 (32.6)	10.7 (6.3) ^#^
Tertiary education	370 (61.5)	9.3 (5.5)
**Received any informative poster or brochure related to CVD**		
Yes	206 (47.3)	10.0 (5.9)
No	317 (52.7)	9.8 (5.9)
**Aware of self-assessment tools that helps to predict CVD risk**		
Yes	205 (34.1)	9.1 (5.6)
No	397 (65.9)	10.3 (6.0) *
**Undergone routine medical check-ups**		
Yes	87	8.75 (5.5) *
No	515	10.10 (6.0)

* *p* < 0.05 compared to counterpart, independent *t*-test; ^#^ *p* < 0.05, ^###^ *p* < 0.001 compared to counterparts, ANOVA.

**Table 2:** Factors associated with INTERHEART mean scores.

Risk Indicator	β	SE	95% Confidence Interval		*p*-Value
			Lower	Upper	
Age	0.034	0.037	−0.038	0.105	0.358
Gender	−3.257	0.478	−4.196	−2.318	<0.001 ***
BMI	0.332	0.055	0.224	0.441	<0.001 ***
Area of residence	−0.256	0.803	−1.832	1.321	0.750
Monthly household income	−0.995	0.570	−2.116	0.125	0.081
Nature of occupation based on level of physical activity	0.460	0.809	−1.128	2.048	0.570
Health-care related occupation	0.157	0.788	−1.390	1.703	0.843
Marital status	−0.525	0.587	−1.678	0.627	0.371
Educational level	−1.040	0.502	−2.026	−0.054	0.039 *

* *p* < 0.05, *** *p* < 0.001, linear regression test. Age and BMI are continuous data; monthly household income, nature of occupation based on level of physical activity, marital status, and educational level have been transformed to binary count.

**Table 3:** Characteristics of the participants according to INTERHEART risk factors, stratified by gender.

Risk Factor (Risk Score)	Total (N = 602)	Male *n* (%) (*n* = 241)	Female *n* (%) (*n* = 361)	*p*-Value (Chi-Square; Male vs. Female)
**Smoking status**				
I never smoked (0)	509 (84.5)	159 (66.0)	350 (97.0)	*p* < 0.001
I am a former smoker (last smoked more than 12 months ago) (2)	40 (6.6)	35 (19.5)	5 (1.4)	
I am a current smoker or I smoked regularly in the last 12 months, and I smoke	53 (8.8)	47 (19.5)	6 (1.7)	
1–5 cigarettes per day (2)	12	10 (21.3)	2 (33.3)	NS
6–10 cigarettes per day (4)	20	17 (36.2)	3 (50.0)	
11–15 cigarettes per day (6)	7	7 (14.9)	0 (0.0)	
16–20 cigarettes per day (7)	12	11 (23.4)	1 (16.7)	
>21 cigarettes per day (11)	2	2 (4.3)	0 (0.0)	
**Second hand smoke**				
**Over the past 12 months, what has been your typical exposure to other people’s tobacco smoke?**				
Less than 1 h or exposure per week or no exposure (0)	277 (46)	112 (46.5)	165 (45.7)	NS
OR One or more hours of second-hand smoke exposure per week (2)	325 (54)	129 (53.5)	196 (54.3)	
Average of hours per week	2.77 h	3.91 h ***	1.62 h	
**Diabetes**				
**Do you have diabetes mellitus?**				
Yes (6)	39 (6.5)	23 (9.5)	16 (4.4)	*p* < 0.05
No or unsure (0)	563 (93.5)	218 (90.5)	345 (95.6)	
**High blood pressure**				
**Do you have high blood pressure?**				
Yes (5)	93 (15.5)	50 (20.7)	43 (11.9)	*p* < 0.01
No or unsure (0)	509 (84.5)	191 (79.3)	318 (88.1)	
**Family history**				
**Have either or both of your biological parents had a heart attack?**				
Yes (4)	130 (21.6)	59 (24.5)	71 (19.7)	NS
No or unsure (0)	472 (78.4)	182 (75.5)	290 (80.3)	
**Waist-to-hip ratio**				
Quartile 1: Less than 0.873 (0)	364 (60.5)	77 (32.0)	287 (79.5)	*p* < 0.001
Quartile 2 and 3: 0.873–0.963 (2)	181 (30)	120 (49.8)	61 (16.9)	
Quartile 4: greater than or =0.964 (4)	57 (9.5)	44 (18.3)	13 (3.6)	
**Psychosocial factors**				
**How often have you felt work or home life stress in the last year?**				
Never or some periods (0)	453 (75.3)	186 (77.2)	267 (74)	NS
OR Several periods of stress or permanent stress (3)	149 (24.8)	55 (22.8)	94 (26)	
**During the past 12 months, was there ever a time when you felt sad, blue, or depressed for two weeks or more in a row?**				
Yes (3)	157 (26.1)	62 (25.7)	95 (26.3)	NS
No (0)	445 (73.9)	179 (74.3)	266 (73.7)	
**Dietary factors**				
**Do you eat salty food or snacks one or more times a day?**				
Yes (1)	199 (33.1)	81 (33.6)	118 (32.7)	NS
No (0)	403 (66.9)	160 (66.4)	243 (67.3)	
**Do you eat deep fried foods or snacks or fast foods 3 or more times a week?**				
Yes (1)	227 (37.7)	107 (44.4)	120 (33.2)	*p* < 0.01
No (0)	375 (62.3)	134 (55.6)	241 (66.8)	
**Do you eat fruit one or more times daily?**				
Yes (0)	413 (68.6)	155 (64.3)	258 (71.5)	NS
No (1)	189 (31.4)	86 (35.7)	103 (28.5)	
**Do you eat vegetables one or more times daily?**				
Yes (0)	413 (68.6)	155 (64.3)	258 (71.5)	NS
No (1)	189 (31.4)	86 (35.7)	103 (28.5)	
**Do you eat meat and/or poultry 2 or more times daily?**				
Yes (2)	366 (60.8)	168 (69.7)	198 (54.8)	*p* < 0.001
No (0)	236 (39.2)	73 (30.3)	163 (45.2)	
**Physical activity**				
**How active are you during your leisure time?**				
I am mainly sedentary or perform mild exercise (requiring minimal effort) (2)	355 (59)	124 (51.5)	231 (64)	*p* < 0.05
OR I perform moderate or strenuous physical activity in my leisure time (0)	247 (41)	117 (48.5)	130 (36)	
**INTERHEART Risk**				
Low risk (Score of 0–9)	329 (54.6)	86 (35.7)	243 (67.3)	*p* < 0.001
Medium risk (Score of 10–15)	171 (28.4)	86 (35.7)	85 (23.5)	
High risk (Score ≥ 16)	102 (16.9)	69 (28.6)	33 (9.1)	

*** *p* < 0.001compared to female, independent *t*-test. NS: Non-significance.

**Table 4:** Characteristics of the participants according to INTERHEART risk factors, stratified by the nature of occupation.

Risk Factor (Risk Score)	Labour Workers (*n*,%) (*n* = 55)	Desk-Bound Workers (*n*,%) (*n* = 288)	*p*-Value (Chi-Square, Manual vs. Office)
**Smoking status**			
I never smoked (0)	34 (61.8)	255 (88.5)	*p* < 0.001
I am a former smoker (last smoked more than 12 months ago) (2)	6 (10.9)	15 (5.2)	
I am a current smoker or I smoked regularly in the last 12 months, and I smoke	15 (27.3)	18 (6.3)	
1–5 cigarettes per day (2)	1 (6.7)	4 (26.7)	*p* < 0.05
6–10 cigarettes per day (4)	5 (33.3)	8 (53.3)	
11–15 cigarettes per day (6)	5 (33.3)	0 (0.0)	
16–20 cigarettes per day (7)	4 (26.7)	2 (13.3)	
>21 cigarettes per day (11)	0 (0.0)	1 (6.7)	
**Second hand smoke**			
**Over the past 12 months, what has been your typical exposure to other people’s tobacco smoke?**			
Less than 1 h or exposure per week or no exposure (0)	31 (56.4)	131 (45.5)	NS (0.139)
OR One or more hours of second-hand smoke exposure per week (2)	24 (43.6)	157 (54.5)	
Average of hours per week	2.86 h ***	1.6 h	-
**Diabetes**			
**Do you have diabetes mellitus?**			
Yes (6)	6 (10.9)	17 (5.9)	NS
No or unsure (0)	49 (89.1)	271 (94.1)	
**High blood pressure**			
**Do you have high blood pressure?**			
Yes (5)	13 (23.6)	40 (13.9)	NS
No or unsure (0)	42 (76.4)	248 (86.1)	
**Family history**			
**Have either or both of your biological parents had a heart attack?**			
Yes (4)	14 (25.5)	59 (20.5)	NS
No or unsure (0)	41 (74.5)	229 (79.5)	
**Waist-to-hip ratio**			
Quartile 1: Less than 0.873 (0)	28 (50.9)	182 (63.2)	NS
Quartile 2 and 3: 0.873–0.963 (2)	20 (36.4)	79 (27.4)	
Quartile 4: greater than or =0.964 (4)	7 (12.7)	27 (9.4)	
**Psychosocial factors**			
**How often have you felt work or home life stress in the last year?**			
Never or some periods (0)	44 (80.0)	200 (69.5)	NS
OR Several periods of stress or permanent stress (3)	11 (20.0)	88 (30.5)	
**During the past 12 months, was there ever a time when you felt sad, blue, or depressed for two weeks or more in a row?**			
Yes (3)	11 (20.0)	81 (28.1)	NS
No (0)	44 (80.0)	207 (71.9)	
**Dietary factors**			
**Do you eat salty food or snacks one or more times a day?**			
Yes (1)	17 (30.9)	101 (35.1)	NS
No (0)	38 (69.1)	187 (64.9)	
**Do you eat deep fried foods or snacks or fast foods 3 or more times a week?**			
Yes (1)	15 (27.3)	113 (39.2)	NS
No (0)	40 (72.7)	175 (60.8)	
**Do you eat fruit one or more times daily?**			
Yes (0)	29 (52.7)	204 (70.8)	*p* < 0.01
No (1)	26 (47.3)	84 (29.2)	
**Do you eat vegetables one or more times daily?**			
Yes (0)	29 (52.7)	204 (70.8)	*p* < 0.01
No (1)	26 (47.3)	84 (29.2)	
**Do you eat meat and/or poultry 2 or more times daily?**			
Yes (2)	36 (65.5)	177 (61.5)	NS
No (0)	19 (34.5)	111 (38.5)	
**Physical activity**			
**How active are you during your leisure time?**			
I am mainly sedentary or perform mild exercise (requiring minimal effort) (2)	29 (52.7)	177 (61.5)	NS
OR I perform moderate or strenuous physical activity in my leisure time (0)	26 (47.3)	111 (38.5)	
**INTERHEART Risk Stratification**			
Low risk (Score of 0–9)	19 (34.5)	167 (58.0)	*p* < 0.001
Medium risk (Score of 10–15)	16 (29.1)	83 (28.8)	
High risk (Score ≥ 16)	20 (36.4)	38 (13.2)	

*** *p* < 0.001, independent *t*-test compared to office-based workers. NS: Non-significance.

**Table 5:** Educational Levels and Occupations Crosstabulation.

Occupation/Educational Level	No formal Education/Primary Education (n)	Secondary Education (*n*)	Tertiary Education (*n*)	Chi-Square, *p*-Value
Labour workers	12	34	9	*p* < 0.001
Desk-bound workers	3	64	221	

## Data Availability

The data presented in this study are available on request from the corresponding authors.

## References

[1] Ministry of Health Malaysia (2017). Clinical Practice Guidelines on Primary and Secondary Prevention of Cardiovascular Disease.

[2] Firus Khan A.Y., Ramli A.S., Abdul Razak S., Mohd Kasim N.A., Chua Y.A., Ul-Saufie A.Z., Jalaludin M.A., Nawawi H. (2022). The Malaysian HEalth and WellBeing AssessmenT (MyHEBAT) Study Protocol: An Initiation of a National Registry for Extended Cardiovascular Risk Evaluation in the Community. Int. J. Environ. Res. Public Health.

[3] Lee S.-K., Ma J., Chan K.Q., Cheong Y., Hong W.-L., Ong R., Tay W., Chua A.L. (2022). Pulse oximeter as a home assessment tool: Knowledge and user experience among the community in Malaysia during the COVID-19 pandemic. Asian Pac. J. Trop. Med..

[4] Badawy M.A.E.M.D., Naing L., Johar S., Ong S., Rahman H.A., Tengah D.S.N.A.P., Chong C.L., Tuah N.A.A. (2022). Evaluation of cardiovascular diseases risk calculators for CVDs prevention and management: Scoping review. BMC Public Health.

[5] McGorrian C., Yusuf S., Islam S., Jung H., Rangarajan S., Avezum A., Prabhakaran D., Almahmeed W., Rumboldt Z., Budaj A. (2011). Estimating modifiable coronary heart disease risk in multiple regions of the world: The INTERHEART Modifiable Risk Score. Eur. Heart J..

[6] Yusuf S., Hawken S., Ôunpuu S., Dans T., Avezum A., Lanas F., McQueen M., Budaj A., Pais P., Varigos J. (2004). Effect of potentially modifiable risk factors associated with myocardial infarction in 52 countries (the INTERHEART study): Case-control study. Lancet.

[7] Philip J., Salim Y., Shun Fu L., Quazi I., Koon T., Sumathy R., Rajeev G., Annika R., Scott A.L., Alvaro A. (2018). Prognostic validation of a non-laboratory and a laboratory based cardiovascular disease risk score in multiple regions of the world. Heart.

[8] Selvarajah S., Kaur G., Haniff J., Cheong K.C., Hiong T.G., van der Graaf Y., Bots M.L. (2014). Comparison of the Framingham Risk Score, SCORE and WHO/ISH cardiovascular risk prediction models in an Asian population. Int. J. Cardiol..

[9] Noor Hassim I., Norazman M.R., Diana M., Khairul Hazdi Y., Rosnah I. (2016). Cardiovascular risk assessment between urban and rural population in Malaysia. Med. J. Malays..

[10] Trautwein E.A., McKay S. (2020). The Role of Specific Components of a Plant-Based Diet in Management of Dyslipidemia and the Impact on Cardiovascular Risk. Nutrients.

[11] Szczepańska E., Białek-Dratwa A., Janota B., Kowalski O. (2022). Dietary Therapy in Prevention of Cardiovascular Disease (CVD)-Tradition or Modernity?. A Review of the Latest Approaches to Nutrition in CVD. Nutrients.

[12] Chiavaroli L., Nishi S.K., Khan T.A., Braunstein C.R., Glenn A.J., Mejia S.B., Rahelić D., Kahleová H., Salas-Salvadó J., Jenkins D.J.A. (2018). Portfolio Dietary Pattern and Cardiovascular Disease: A Systematic Review and Meta-analysis of Controlled Trials. Prog. Cardiovasc. Dis..

[13] Macho-González A., Garcimartín A., López-Oliva M.E., Bastida S., Benedí J., Ros G., Nieto G., Sánchez-Muniz F.J. (2020). Can Meat and Meat-Products Induce Oxidative Stress?. Antioxidants.

[14] Siew-Keah L., Hann K., Hoe S., En L., Chin L., Chan M., Kang T., Ang-Lim C. (2022). Awareness and perceived barriers in practicing healthy living to prevent hypertension among young adults in Malaysia. Asian Pac. J. Trop. Med..

[15] Ministry of Health Malaysia (2019). National Health and Morbidity Survey 2019 Non-Communicable Diseases, Healthcare Demand, and Health Literacy.

[16] Lim K.H., Lim H.L., Teh C.H., Kee C.C., Heng P.P., Cheah Y.K., Mohd Ghazali S. (2018). Secondhand smoke (SHS) exposure at home and at the workplace among non-smokers in Malaysia: Findings from the Global Adult Tobacco Survey 2011. Tob. Induc. Dis..

[17] Barnoya J., Glantz S.A. (2005). Cardiovascular effects of secondhand smoke: Nearly as large as smoking. Circulation.

[18] Wu X., Zhu B., Xu S., Bi Y., Liu Y., Shi J. (2020). A cross country comparison for the burden of cardiovascular disease attributable to tobacco exposure in China, Japan, USA and world. BMC Public Health.

[19] U.S. Department of Health and Human Services (2014). The Health Consequences of Smoking—50 Years of Progress: A Report of the Surgeon General.

[20] Kaufmann R., Babb S., O’Halloran A., Asman K., Bishop E., Tynan M., Caraballo R., Pechacek T., Bernert J., Blount B. (2010). Vital signs: Nonsmokers’ exposure to secondhand smoke-United States, 1999–2008. Morb. Mortal. Wkly. Rep..

